# Effect of End Groups on the Raman Spectra of Lycopene and *β*-Carotene under High Pressure

**DOI:** 10.3390/molecules16031973

**Published:** 2011-02-25

**Authors:** Ming-Ming Huo, Wei-Long Liu, Zhi-Ren Zheng, Wei Zhang, Ai-Hua Li, Da-Peng Xu

**Affiliations:** 1 Center for Condensed Matter Science and Technology, Department of Physics, Harbin Institute of Technology, Harbin, 150001, China; E-Mails: hithuomm@163.com (M.-M.H.); zhang_wei_hit@126.com (W.Z.); aihua.li@126.com (A.-H.L.); 2 College of Physics, Ji Lin University, Changchun 130023, China; E-Mail: xudp@jlu.edu.cn

**Keywords:** pressure, structure, Raman, lycopene, *β*-carotene

## Abstract

The Raman spectra of all-*trans-*lycopene in *n*-hexane were measured under high pressure, and the results compared with those of *β-*carotene. The different pressure effects on Raman spectra are analyzed taking into account the different structures of lycopene and *β-*carotene molecules. It is concluded that: (a) the vibronic coupling between the *S*_1_ and *S*_0_ states of *β*-carotene is stronger than that of lycopene, (b) the diabatic frequency increment of the ν_1 _mode is more susceptible to pressure than that of the ν_2_ mode for lycopene, and (c) *β*-rings rotation can relieve the pressure effect on the C=C bond length in *β-*carotene. This work provides some insights for elucidating the structural and environmental effects on Raman spectra of carotenoids.

## 1. Introduction

Carotenoids play important roles in photobiology, participating in a variety of functions, including antioxidation, light-harvesting, photoprotection [1,2], and so on. Knowledge of the spectroscopic characteristics of the carotenoids’ molecular configuration, excited electronic states, and solvent effect are necessary prerequisites for understanding their functions in complex natural systems [2]. 

Generally speaking, differences in molecular structure may greatly affect the biological functions of carotenoids. A carotenoid molecule consists of three segments: a relatively rigid conjugated backbone and two end groups. The end groups can usually rotate easily around the bonds connecting them to the conjugated chain [3]. The physical and chemical properties of carotenoids are immediately affected by their molecular structures. For example, lycopene and *β*-carotene are two typical carotenoids, both of which have 11 *π*-conjugated double bonds. The only difference between them is the end groups of the polyene backbone – lycopene has an open-chain structure while *β*-carotene has a *β*-ring at each end [4]. The presence of the terminal *β*-rings markedly affects internal conversion process [5] and energy transfer reactions of carotenoids [6]. Furthermore, with the help of *β*-rings, *β*-carotene has a more high-laying triplet state, higher rotational barriers and lower rotational rates than lycopene [7].

Raman spectroscopy has been used extensively to identify the species, content and distribution of carotenoids in biological systems for its advantages of rapid sampling and analysis [8,9]. The length of the polyene chain, the terminal groups of carotenoids and their interactions with other plant constituents can give rise to small shifts in the vibrational frequencies of the main characteristic Raman bands [10]. For these reasons, the investigations of environmental and structural effects on Raman spectra can offer important insights for identifying the carotenoids in biological systems. High pressure can amplify some intermolecular and intramolecular interactions making them easier to investigate [11], therefore high-pressure Raman spectra have been widely used in the study of both inorganic and organic materials [12,13]. In order to illustrate the environmental and structural effects on Raman spectra of carotenoids, in this work, we measured high-pressure Raman spectra of lycopene and compared the results to those obtained for *β*-carotene.

## 2. Results and Discussion

### 2.1. Effects of end groups on Raman spectra of carotenoids at ambient conditions

**Figure 1:**
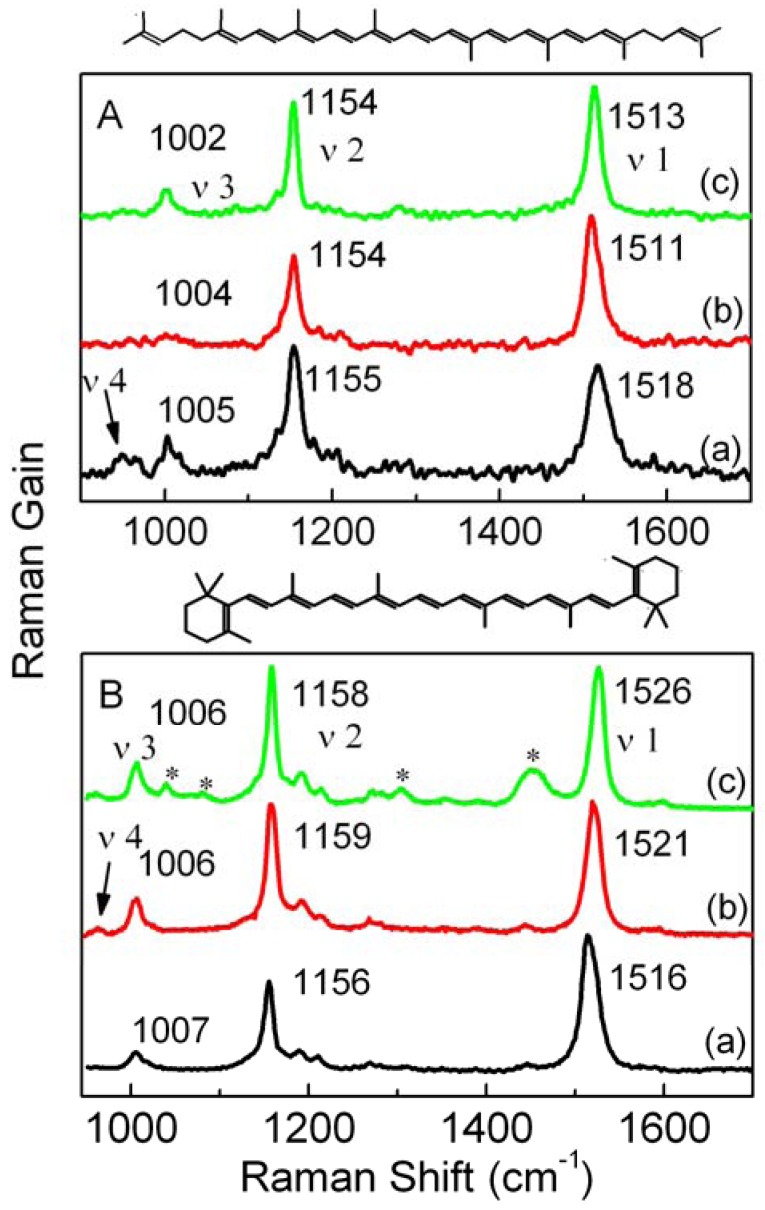
Raman spectra of lycopene (A) and *β*-carotene (B) in the solid state (a), CS_2_ (analytical grade) (b) and *n*-hexane (c) under ambient conditions. Asterisks in panel B(c) denote the Raman bands from *n*-hexane.

Raman spectra of lycopene in the solid state and in CS_2 _and *n*-hexane at ambient conditions are shown in Figure 1A. The Raman spectrum of crystalline lycopene is composed of three main bands, which are located at 1,518, 1,155, and 1,005 cm^−1^ and called the *ν*_1_, *ν*_2_, and *ν*_3_ bands, respectively. Normal coordinate analyses have assigned these three bands to C=C in-phase stretching, C-C stretching, and methyl in-plane rocking modes, respectively [14]. The weak band around 960 cm^−1^ in the solid state is called the *ν*_4_ band and is assigned to the C-H out-of-plane wagging [14]. 

As shown in Figure 1A, the *ν*_1 _band of lycopene shifts to lower frequencies in CS_2 _and *n*-hexane compared with those in the solid state; the ν_4 _band can be seen clearly in the solid state, but it does not appear in solution. It is well-known that the *ν*_1 _frequency is relevant to the effective conjugated length of a carotenoid [15,16], and the relative intensity of the *ν*_4_ band is determined by the degree of deviation from the conjugated plane [14], therefore the visible *ν*_4 _band and higher frequency of the *ν*_1 _band for lycopene in the solid state can be ascribed to the conformational distortion. The case of *β*-carotene is just the opposite, as shown in Figure 1B, which suggests that the conformational distortion in solution are greater than that in the solid state.

It can be seen from Figure 1 that the frequency difference between *ν*_1_ modes in *n*-hexane and CS_2_ is larger for *β*-carotene than that for lycopene; this is related to the different vibronic coupling between the *S*_1_ and *S*_0_ states. Vibronic coupling in polyenes was first proposed to account for the unexpected differences between the transition frequencies of the C=C stretching mode in the *S*_0_ and *S*_1_ states [17]. The vibronic-coupling theory has been applied to explain the *S*_1_ state Raman spectrum of *β-*carotene [5,18]. On the basis of this theory, a pressure-relevant competitive mechanism has been proposed by Liu *et al.* [19] to explain the diverse pressure effects on the Raman frequency of the *ν*_1_ and *ν*_2_ bands of *β-*carotene in different solvents. In this model, the adiabatic frequency of the *i*th vibration mode in the *S*_0_ state can be expressed as [19]:

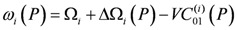
(1)
where 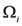 is the diabatic frequency of the *i*th vibration mode in the *S*_0_ state at normal pressure, 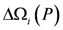 is the increment of 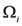 as a result of the shortened bond lengths induced by high pressure, 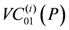 is the contribution of vibronic coupling. Consequently, the pressure induced increase of the adiabatic frequency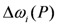 can be expressed as:

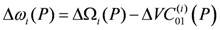
(2)
where 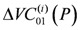 is the increment of 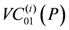. It has been shown that stronger vibronic coupling between the *S*_0_ and *S*_1_ states can result in a more obvious solvent effect on the *ν*_1_ frequency [19,20]. For these reasons, the larger difference between *ν*_1_ frequency in *n*-hexane and CS_2_ for *β*-carotene than that for lycopene suggests that the vibronic coupling between the *S*_1_ and *S*_0_ states of *β*-carotene is stronger than that of lycopene.

### 2.2. Effects of end groups on Raman spectra of carotenoids under high pressure

**Figure 2:**
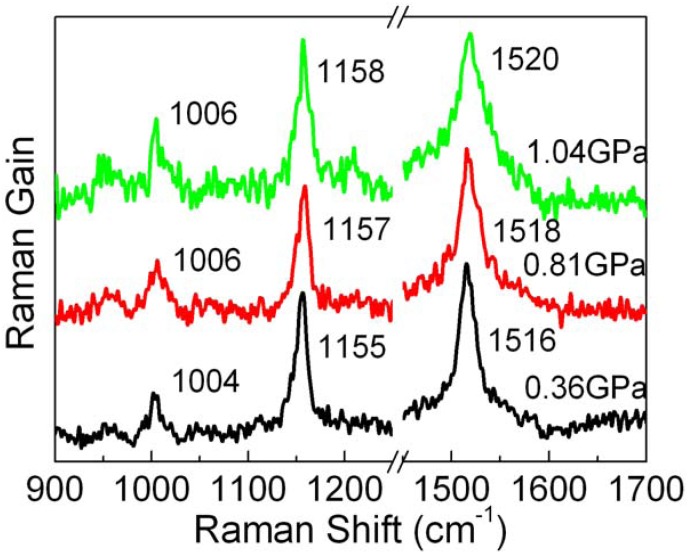
Raman spectra of lycopene in *n*-hexane under different pressures.

Several representative Raman spectra of lycopene in *n*-hexane under high pressure are shown in Figure 2. As the pressure increases, the major Raman bands move to higher wave numbers. 

**Figure 3:**
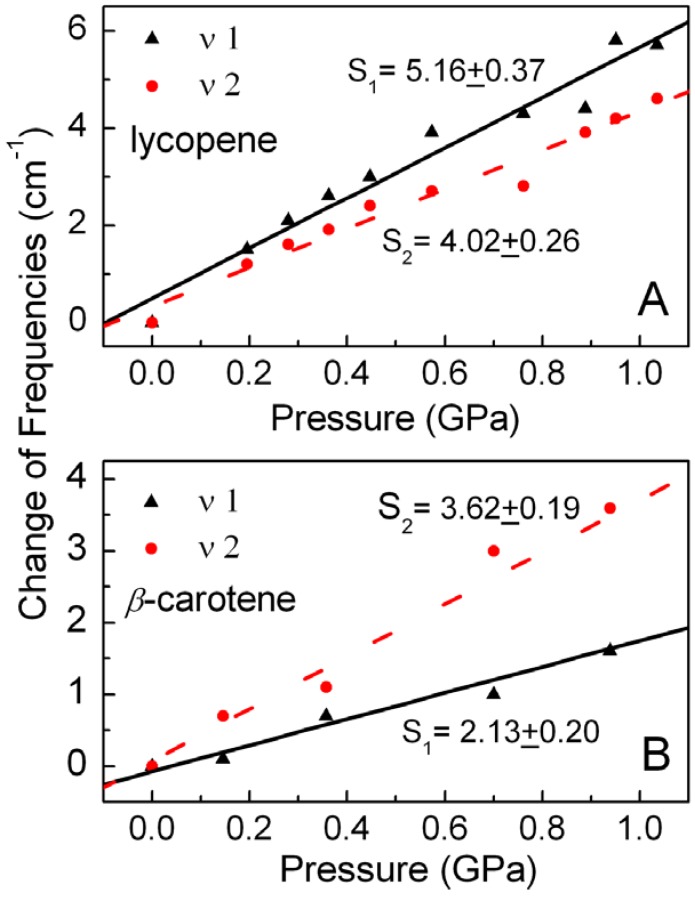
Effect of pressure on Raman frequencies of (A) lycopene and (B) *β-*carotene in *n*-hexane. The changes in the Raman frequencies relative to ambient pressure are shown for the ν_1 _(▲) and ν_2 _(•) bands. The slopes (S) obtained from liner fitting are also presentedwith frequency in cm^−1^ and pressure in GPa.

A Lorentzian curve fitting routine was used to determine the center frequency of the Raman bands. In order to systematically analyze the effects of different terminal structures on the Raman spectra of carotenoids under high pressure, the *ν*_1_ and *ν*_2_ frequencies of lycopene and *β-*carotene in *n*-hexane are plotted versus pressure in Figure 3. Contrary to the case for *β-*carotene [19], the rate of increase of the ν_1_ frequency is faster than that of the ν_2_frequency for lycopene. The ν_1 _frequency of lycopene increases faster than that of *β*-carotene, while the ν_2 _frequency of lycopene has almost the same rate of increase as that of *β*-carotene. These results can be explained by using the pressure-relevant competitive mechanism [19] taking into account the different end groups of lycopene and *β-*carotene molecules.

Frequencies of ν_1 _and *ν*_2 _bands increase with the pressure increase in *n*-hexane [19], because the contribution of pressure on the diabatic frequency is greater than that on vibronic coupling, *viz*.:

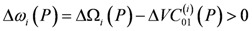
(3)

For the *ν*_2_ band, the contribution of vibronic coupling is absent [19], so at first view, the rate of increase of the *ν*_2_ frequency should be faster than that of the *ν*_1_ frequency, but Figure 3A shows that for lycopene the rate of increase of the ν_1_ frequency is faster than that of the *ν*_2 _band. It can therefore be concluded from Eq. (2) that although vibronic coupling between the *S*_0_ and *S*_1_ states of lycopene slows down the increasing rate of ν_1 _frequency, the total contribution of the diabatic frequency and vibronic coupling to the ν_1 _mode is still larger than the single contribution of the diabatic frequency to the ν_2 _mode, *viz*.:

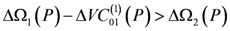
(4)

This suggests that the C=C stretching vibration is more sensitive to pressure, which is inconsistent with the general idea that double bonds usually involve more energy than single bonds and so the bond length of C=C bond is less affected by high pressure than C-C bonds. This can be explained by taking account of the special electronic structure of conjugated polyene system. It is well-known that the C-C bond consists of only a localized σ bond, which makes up the molecule skeleton of a conjugated system, whereas C=C bond consists of a σ bond and a π bond, which is delocalized and responsible for the major part of the C=C stretching frequency. π bonds are vertical to the plane of polyene skeleton, so they are more sensitive to solvent (or pressure) than σ bonds. It can therefore be seen from Eq. (4) that although the bond length of a C=C bond is less affected by high pressure than the C-C bond, the diabatic frequency increment of the ν_1 _mode is more susceptible to pressure than that of the ν_2 _mode.

It can be seen from Figure 3 that the rate of increase of the *ν*_1_ frequency (S_1_ = 5.16 ± 0.37) for lycopene is faster than that (S_1_ = 2.13 ± 0.20) for *β*-carotene. This can be easily explained according to Eq. (2). On the one hand, the lycopene molecule has no *β*-rings, so the C=C bonds in lycopene can obviously be compressed more under high pressure as compared with those in *β*-carotene, since the *β*-ring rotation in *β*-carotene can relieve the pressure effect on the C=C bond lengths. On the other hand, *β*-ring rotation can reduce the dihedral angles between the conjugated polyene plane and the *β*-rings, and depress the ν_1_ frequency [21]. It can be found from these two factors that 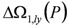
>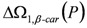. In addition, Figure 3 indicates that with increasing pressure, vibronic coupling between *S*_0_ and *S*_1_ of lycopene is smaller than that of *β*-carotene, namely, 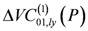
<
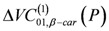
. Therefore:

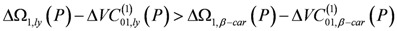
(5)
*viz*. the *ν*_1_ frequency increases faster for lycopene than in *β*-carotene.

The increase of the ν_2_frequency merely depends on the equilibrium geometry at a given pressure, and is independent of vibronic coupling [19], so it is reasonable that the ν_2 _frequency of lycopene (S_2 _= 4.02 ± 0.26) has almost the same rate of increase as that of *β*-carotene (S_2 _= 3.62 ± 0.19), as shown in Figure 3.

## 3. Experimental

### General

Lycopene was purchased from Shanghai Shunbo Biological Engineering Technology and stored at −20 °C in the dark. In order to obtain better Raman signals, excess lycopene was dissolved in *n*-hexane (HPLC grade) to achieve a saturated solution. In all experiments, fresh solution was used immediately after its preparation to avoid degeneration. No crystals precipitated during the measurements under high pressure.

Quasi-hydrostatic pressure was applied to the sample using a sapphire anvil cell. Two sapphires with 1.5 mm culet faces were used. The gasket was made of copper sheet with an initial thickness of 0.8 mm and had a hole with a diameter of 0.8 mm. The pressure in the cell was determined by observing the shift of fluorescence from a small ruby contained within the sample cell [21]. 

Both Raman spectra and ruby fluorescence measurements were performed with a Raman microscope (Jobin Yvon, HR800) equipped with an Ar^+^ laser (514.13 nm) and a multiple track CCD detector. The laser power was 20 mW and the integration time was 10 s. For both the normal and high pressure experiments, the instrumental resolution was 1 cm^−1^, and therefore, the error of pressure calibration was ±0.07 GPa [19,21].

## 4. Conclusions

In this work, we measured the Raman spectra of lycopene under high pressure, and the results were compared with those of *β*-carotene. The high-pressure Raman spectra of lycopene suggest that the diabatic frequency increment of the ν_1 _mode is more susceptible to pressure than that of the ν_2 _mode, so the rate of increase of the *ν*_1 _band frequency is faster than that of the *ν*_2 _band frequency. The greater pressure effect on the *ν*_1_ frequency for lycopene than for *β*-carotene can be ascribed to the *β*-ring rotation, which relieves the pressure effect on the C=C bond length. This work opens a new pathway for elucidating the structural and environmental effects on the Raman spectra of carotenoids.
